# Strangulated Bowel Obstruction Due to Hiatal Hernia After Laparoscopic Total Gastrectomy

**DOI:** 10.7759/cureus.58610

**Published:** 2024-04-19

**Authors:** Hirohito Kakinuma, Michitaka Honda, Takumi Funo, Ryutaro Mashiko, Yoshinao Takano

**Affiliations:** 1 Department of Minimally Invasive Surgical and Medical Oncology, Fukushima Medical University, Fukushima, JPN; 2 Department of Surgery, Southern Tohoku Research Institute for Neuroscience, Southern Tohoku General Hospital, Koriyama, JPN

**Keywords:** roux-en-y reconstruction, internal hernia, laparoscopic total gastrectomy, strangulated bowel obstruction, esophageal hiatal hernia

## Abstract

Laparoscopic total gastrectomy results in more internal hernias than open surgery. However, there are few reports of incarcerated hiatal hernia after laparoscopic total gastrectomy. Here, we report a case of a 79-year-old male who underwent urgent surgical intervention for a strangulated intestinal obstruction due to an incarcerated hernia through the esophageal hiatus following laparoscopic total gastrectomy. In this case, an esophageal hiatal hernia was present before gastrectomy, but was not repaired. Additionally, the patient experienced significant weight loss after gastrectomy. Preoperative hiatal hernia and marked postoperative weight loss may pose risks.

## Introduction

Laparoscopic surgery is preferred over open surgery because it results in fewer postoperative adhesions [1], thus reducing the incidence of postoperative adhesive intestinal obstruction. However, it is also believed to increase the development of internal hernias [2]. Internal hernias following laparoscopic total gastrectomy (LTG) and Roux-en-Y (RY) reconstruction occur at a frequency of 4.9% [3], and often occur at the jejunal mesenteric defects, Petersen’s defect, and mesocolic defect of the transverse colon route [4]. Among these, herniation through the esophageal hiatus is rare, at 0.7% [5]. Diaphragmatic crus incisions not accompanied by repair after gastric cancer surgery are considered a risk factor for esophageal hiatal hernia [6], but other risk factors have not been clearly identified. This report describes an emergency surgical intervention for a case of strangulated intestinal obstruction due to an incarcerated hernia through the esophageal hiatus following LTG.

## Case presentation

**Figure 1:**
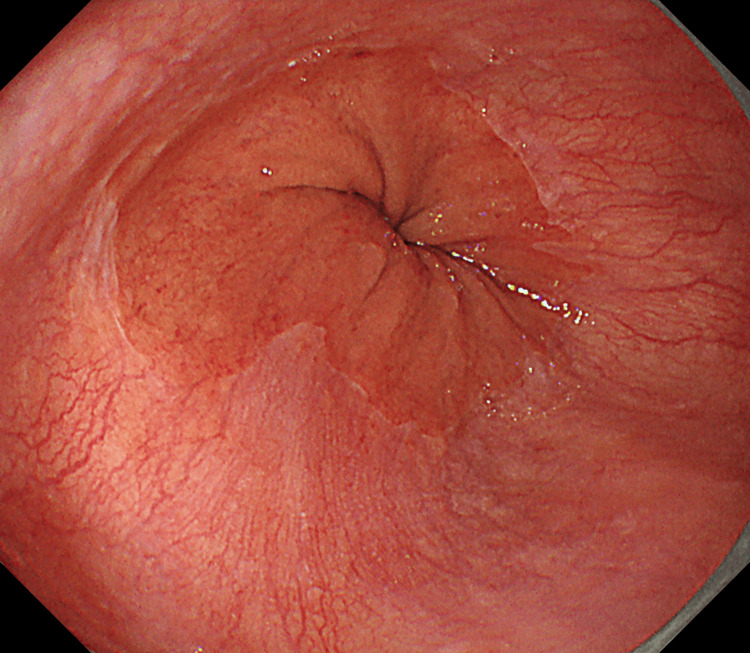
The gastroesophageal junction was deviated orally from the esophageal hiatus.

**Figure 2:**
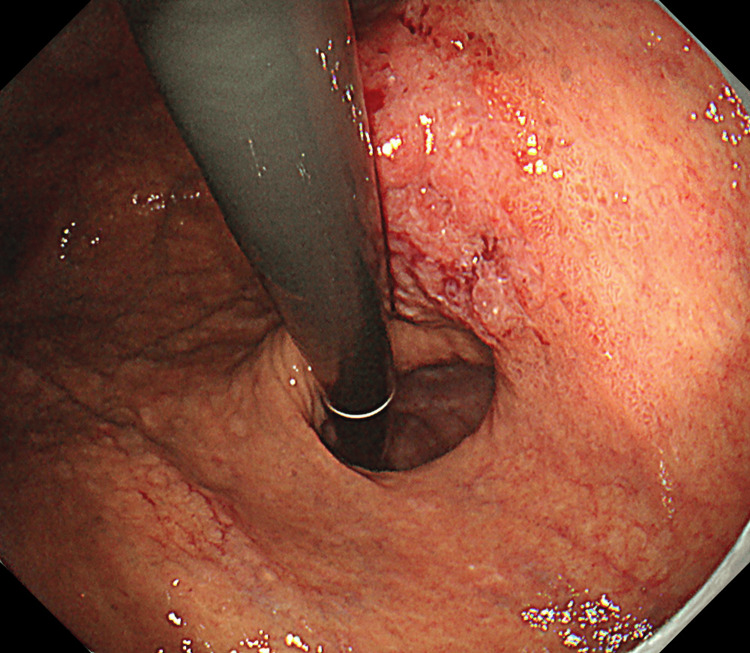
In addition to the dilation of the esophageal hiatus, the esophageal mucosa was visible through the gap at the cardia.

**Figure 3:**
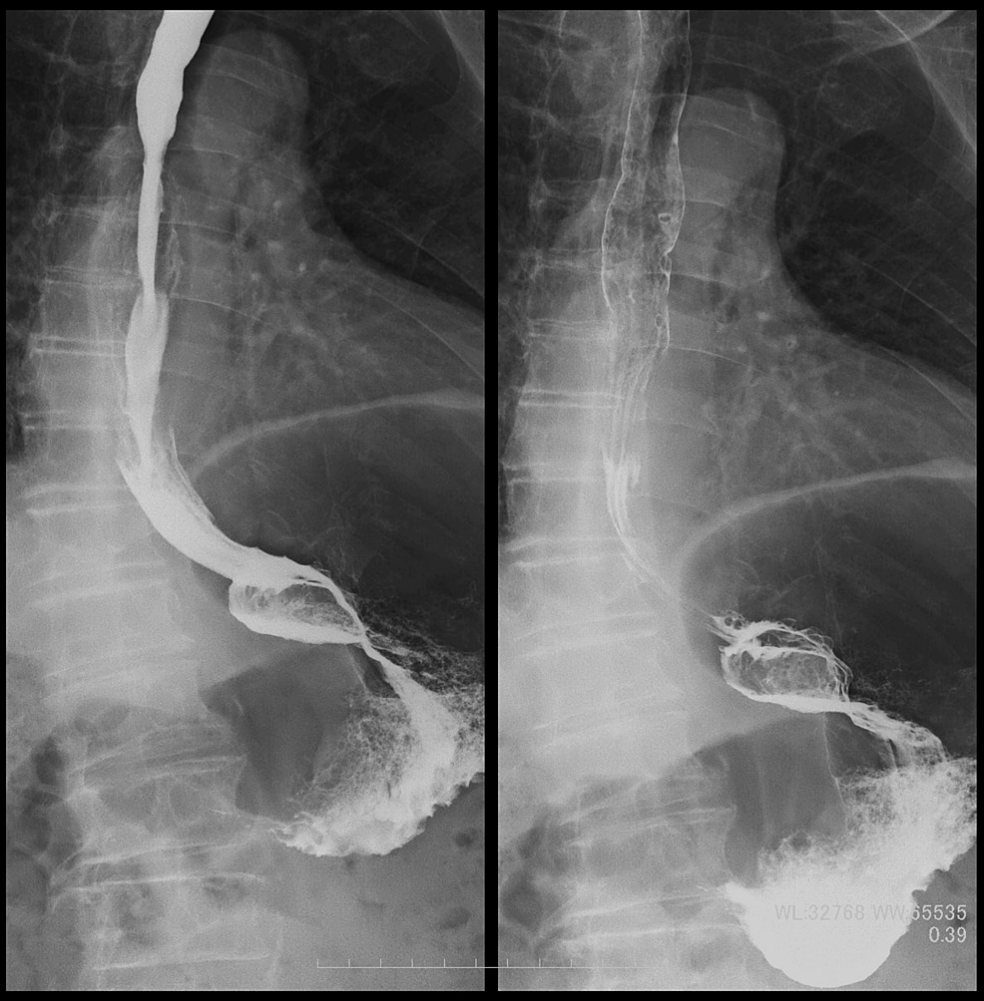
The gastroesophageal junction was displaced orally from the esophageal hiatus.

**Figure 4:**
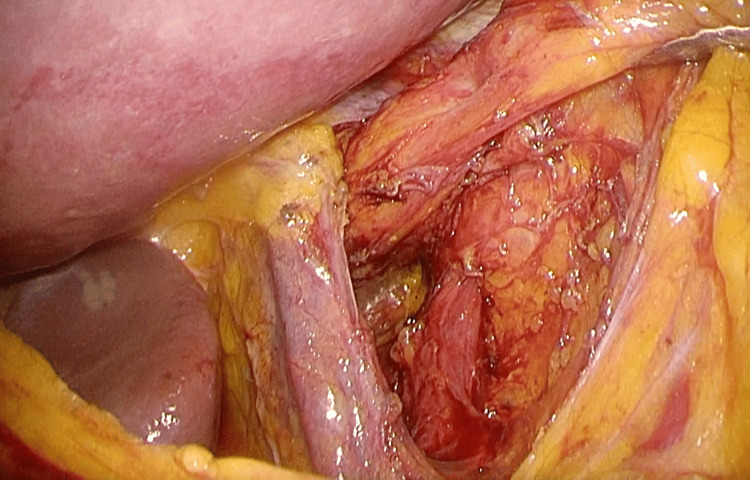
Enlargement of the esophageal hiatus was noted.

A 79-year-old male patient presented to our emergency department with postprandial epigastric pain. He had undergone LTG, D2 lymphadenectomy, and RY reconstruction 19 months previously for gastric cancer. Pathologically, the cancer was classified as T3N1M0 stage IIB based on TNM (tumor, node, metastasis) classification. Three months postoperatively, liver metastasis recurrence was observed, and the patient was treated as an outpatient with S-1. Pre-gastrectomy upper gastrointestinal endoscopy and upper gastrointestinal series (Figures 1-3) showed that the gastroesophageal junction was displaced orally from the esophageal hiatus, with a mild sliding-type esophageal hiatal hernia (Grade I). There were no symptoms or endoscopic findings of reflux esophagitis, and cruroplasty was not performed during gastric cancer surgery (Figure 4). Diaphragmatic crus incision and lower mediastinal lymph node dissection were not performed intraoperatively. After the gastrectomy, the patient was unaware of any chronic symptoms.

**Figure 5:**
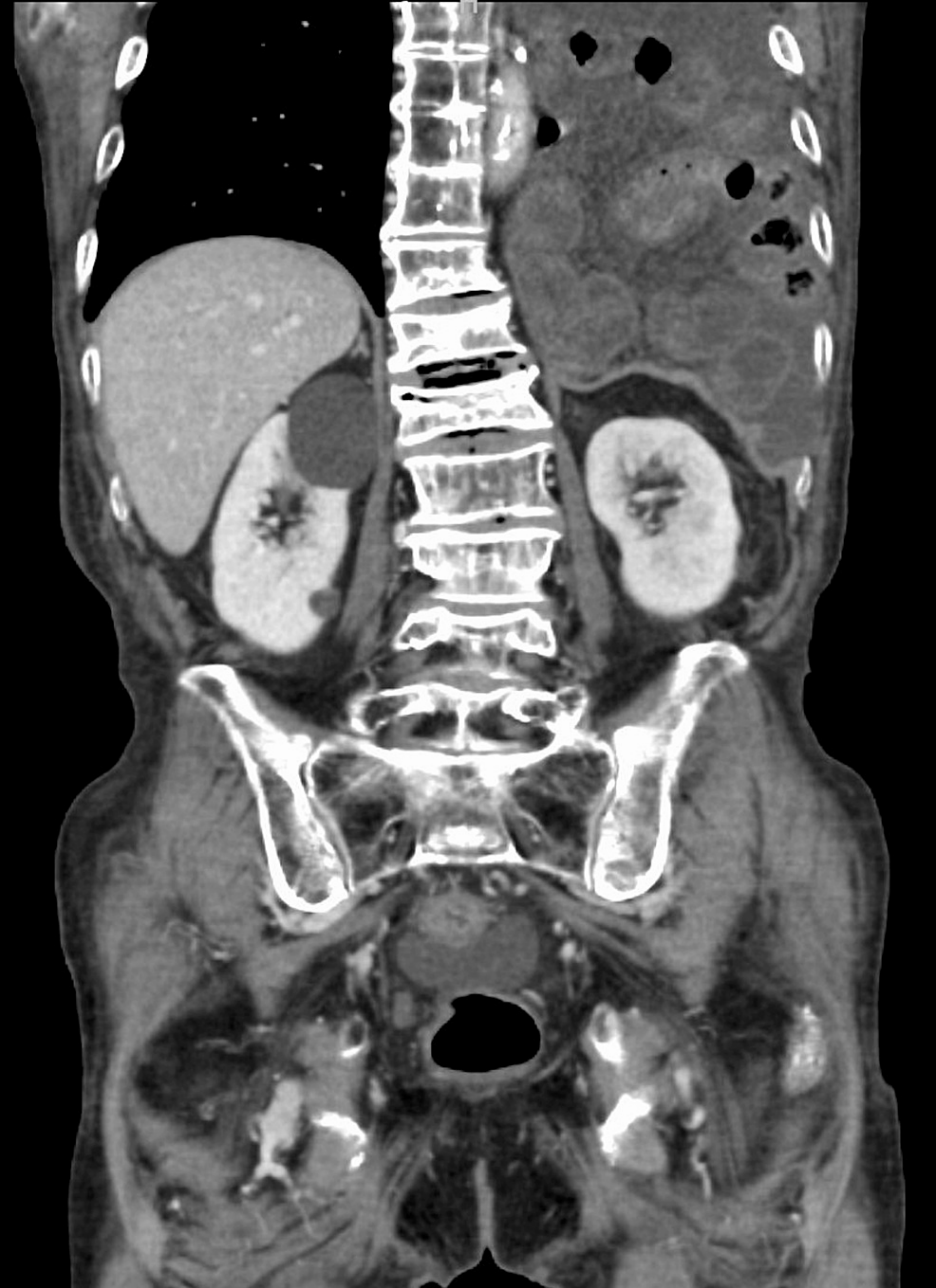
The dilated small intestine had herniated into the thoracic cavity. Part of the intestinal wall showed poor contrast enhancement, and congestion of the mesentery was noted.

At presentation, the patient’s vital signs were stable. Spontaneous epigastric pain and muscular guarding were also observed. Blood tests revealed hyperlactatemia (lactate 3.6 mol/L) without signs of an inflammatory response. Thoracoabdominal contrast-enhanced CT showed that the small intestine extended through the esophageal hiatus into the thoracic cavity. Contrast enhancement of the intestinal wall was poor, and mesenteric congestion was observed, raising suspicion of strangulation of the herniated small intestine (Figure 5). The patient was diagnosed with a strangulated intestinal obstruction due to an esophageal hiatal hernia and underwent emergency surgery. 

**Figure 6:**
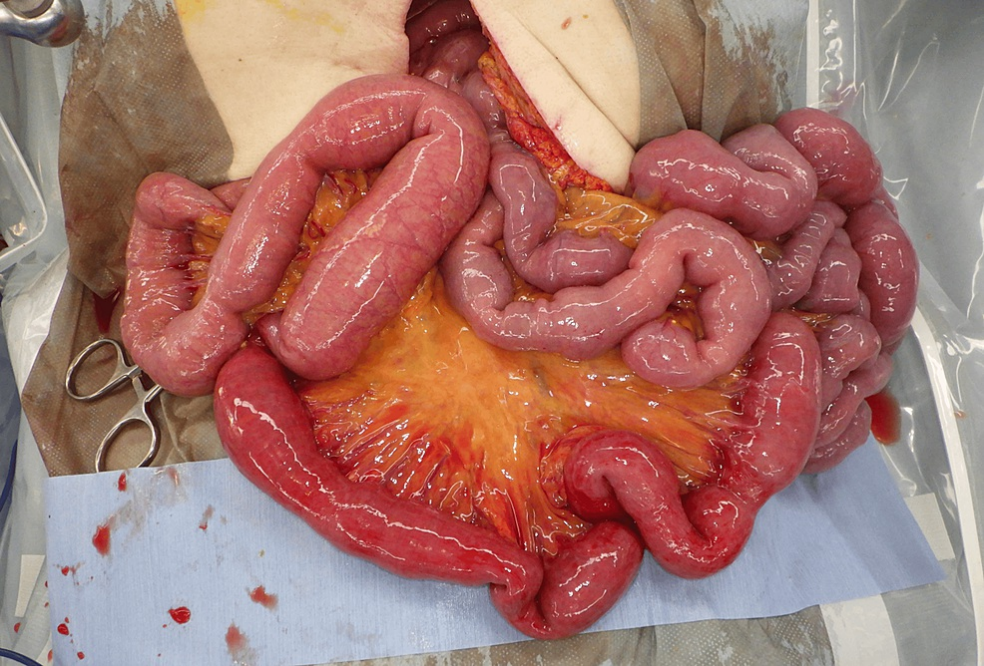
The strangulated small intestine had turned a dark red color, but there were no signs of necrosis.

**Figure 7:**
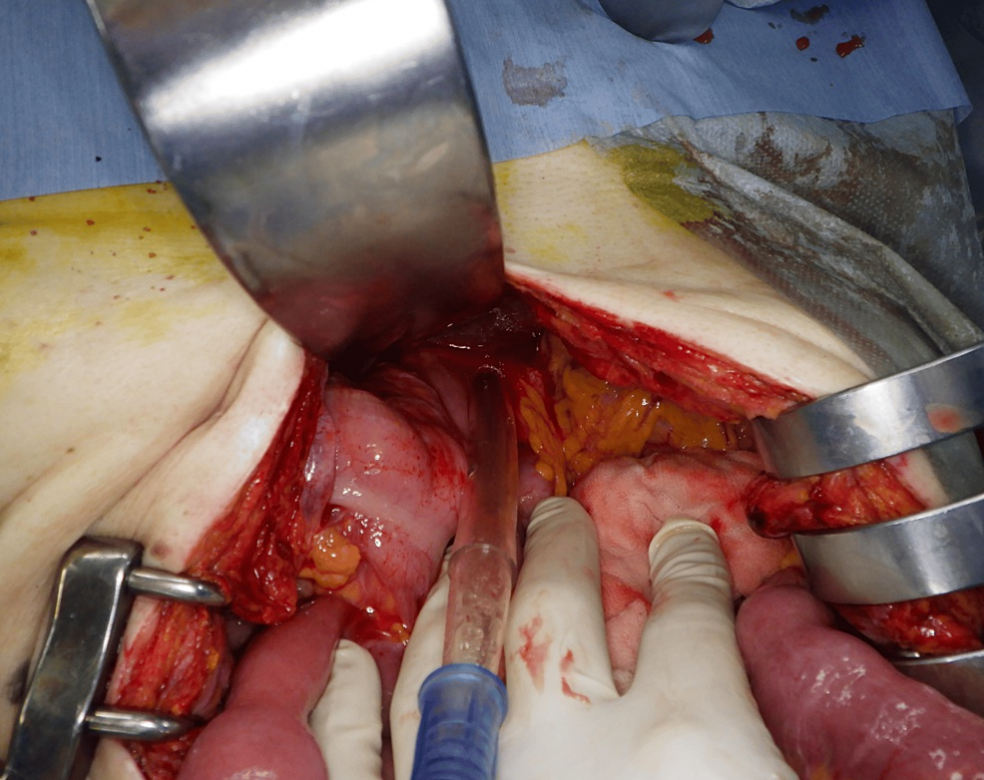
The hernia orifice was the esophageal hiatus, which measured approximately 2 cm (the site at which the suction tube inserted is the hernia orifice).

The patient visited during the night time, and surgeons skilled in laparoscopy were not available at the time. Therefore, we chose the open surgery. Upon midline laparotomy and inspection of the abdominal cavity, the esophageal hiatus acted as a hernial orifice, with the small intestine herniating into the left thoracic cavity. No adhesions were observed in the thoracic or abdominal cavities and the small intestine was reduced back into the abdominal cavity. Approximately 40 cm of the herniated intestine showed congestion; however, there were no signs of necrosis (Figure 6). The hernial orifice was approximately 2 cm in size (Figure 7). The hernia sac was not excised, but the hernial orifice was sutured closed with three non-absorbable stitches, concluding the surgery. The patient recovered uneventfully and was discharged on the fifth postoperative day.

## Discussion

We reported a case of internal hernia with the esophageal hiatus as a hernial orifice after LTG with RY reconstruction, which required emergency surgery.

LTG for gastric cancer has become widely accepted owing to its equivalent rate of postoperative complications compared to open surgery [7] and the benefit of shorter hospital stays [8]. Laparoscopic surgery is associated with fewer postoperative adhesions, which reduces the incidence of adhesive intestinal obstruction [1]. However, there is a reported increase in the incidence of internal hernias [9]. Internal hernia following LTG with RY reconstruction often occurs at the jejunal mesenteric defects, Petersen’s defect, and mesocolic defect of the transverse colon route, with closure of the mesenteric defects being crucial [4].

Reports on the closure of mesenteric defects, especially Petersen's defects, are numerous, indicating the importance of this practice [10]. Nonetheless, internal hernias emanating from the esophageal hiatus after total gastrectomy are rarely reported. In a Japanese retrospective study of 8,938 patients who underwent gastrectomy for gastric cancer, only 13 (0.19%) were diagnosed with internal hernias, with only one case (0.01%) considered to be an esophageal hiatal hernia [9]. To date, six cases of strangulated intestinal obstruction due to esophageal hiatal hernia incarceration have been reported [11-16]. Including this case, we present seven cases in Table 1 (modified from Itamoto et al. [16]). All cases were male. The reason may be that, in general, the incidence of gastric cancer is higher in males, which we attribute to reflected trends. Six cases occurred after total gastrectomy, and one occurred after proximal gastrectomy. The onset varied from two days to 21 months postoperatively, with four cases developing after laparoscopic surgery. Diaphragmatic crus incisions were made in five cases during gastric cancer surgery, of which only one underwent cruroplasty at the esophageal hiatus. Two patients, including the present case, had recognized esophageal hiatal hernias preoperatively; neither patient had undergone cruroplasty.

**Table 1:** Summary of a total of seven cases of incarcerated esophageal hiatal hernia OTG, open total gastrectomy; LTG, laparoscopic total gastrectomy; RY, Roux-en-Y reconstruction; HALS, hand-assisted laparoscopic surgery

No	Author	Age	Sex	Primary surgery	Hiatal hernia before gastrectomy	Crus incision and repair in primary surgery	Interval	Symptoms	Incarcerated organ	Surgical approach	Surgical procedure
1	Murata et al., 2013 [11]	44	M	OTG RY	No	Yes/Yes	2 days	Dyspnea and chest pain	Transverse colon	Open	Close the hernia defect
2	Tashiro et al., 2016 [12]	78	M	OTG RY	No	Yes/No	2 days	None	Transvers colon・Jejunal limb	Open	Close the hernia defect
3	Santos et al., 2016 [13]	76	M	LTG RY	No	No/No	2 months	Vomiting and abdominal pain	Small intestine	Laparoscopic	Resect ischemic lesion with enteric anastomosis and close the hernia defect
4	Wang et al., 2019 [14]	76	M	LTG RY	No	Yes/No	5 days	Abdominal fullness, vomiting, dyspnea, and high fever	Jejunal limb	Open	Resect ischemic lesion with enteric anastomosis and close the hernia defect
5	Ezzy et al., 2021 [15]	66	M	OTG RY	Yes	unknown/unknown	12 months	Vomiting and abdominal pain	Small intestine	Open	Close the hernia defect
6	Itamoto et al., 2023 [16]	66	M	LPG Double tract	Unknown	Yes/No	18 months	Vomiting and abdominal pain	Transverse colon	HALS	Close the hernia defect
7	Present case	79	M	LTG RY	Yes	No/No	21 months	Vomiting and abdominal pain	Small intestine	Open	Close the hernia defect

Non-repaired diaphragmatic crus incisions after gastric cancer surgery are considered a risk factor for esophageal hiatal hernia [6], although other risk factors have not been clarified. Risk factors for esophageal hiatal hernias following esophageal cancer surgery include diaphragmatic crus incisions, pre-existing esophageal hiatal hernias, minimally invasive surgery, and postoperative weight loss [17]. In the current case, the esophageal hiatal hernia was recognized before gastrectomy, but as asymptomatic hiatal hernias are generally not deemed necessary to repair [18], it was left unrepaired. Moreover, the patient had a significant weight loss of 13.5 kg (26.0%) in comparison to before gastric cancer surgery, with his BMI dropping from 24.2 to 18.8 kg/m^2^. This suggests that generalized muscle weakening and a decrease in intra-abdominal fat might have enlarged the esophageal hiatus, thereby increasing the risk of intestinal herniation. Itamoto et al [16] similarly reported that weight loss could be the risk factor for esophageal hiatal hernia. Additionally, the absence of adhesions due to laparoscopic surgery may have further increased the risk of internal hernias.

Preventive measures for esophageal hiatal hernias after esophagectomy have been proposed, such as closing the hiatus with non-absorbable sutures, avoiding diaphragmatic crus incisions, and anchoring the reconstructed gastric tube to the diaphragm [19,20]. Similarly, in LTG, cruroplasty may prevent postoperative internal hernias. It might be prudent to perform cruroplasty in patients with preoperative esophageal hiatal hernias regardless of symptoms. The prevention of postoperative weight loss is crucial.

## Conclusions

The risk factors for incarcerated esophageal hiatal hernia after gastric cancer surgery have not been clearly identified. In this case, an esophageal hiatal hernia was present before gastrectomy, but was not repaired. Additionally, the patient experienced significant weight loss after gastrectomy. Preoperative hiatal hernia and marked postoperative weight loss may pose risks. As a preventative measure, suturing of the diaphragmatic crura at the esophageal hiatus should be considered during gastrectomy. The prevention of postoperative weight loss is also important.
